# Diagnosis accuracy of PCA3 level in patients with prostate cancer: a systematic review with meta-analysis

**DOI:** 10.1590/S1677-5538.IBJU.2019.0360

**Published:** 2020-07-31

**Authors:** Zhiqiang Qin, Jianxiang Yao, Luwei Xu, Zheng Xu, Yuzheng Ge, Liuhua Zhou, Feng Zhao, Ruipeng Jia

**Affiliations:** 1 Nanjing Medical University Nanjing First Hospital Department of Urology Nanjing China Department of Urology, Nanjing First Hospital, Nanjing Medical University, Nanjing, China; 2 Huzhou first people's hospital Department of Urology Huzhou China Department of Urology, Huzhou first people's hospital, Huzhou, China

**Keywords:** prostate cancer antigen 3, human [Supplementary Concept], Prostate cancer, familial [Supplementary Concept], Meta-Analysis [Publication Type]

## Abstract

**Background:**

The diagnostic value and suitability of prostate cancer antigen 3 (PCA3) for the detection of prostate cancer (PCa) have been inconsistent in previous studies. Thus, the aim of the present meta-analysis was performed to systematically evaluate the diagnostic value of PCA3 for PCa.

**Materials and Methods:**

A meta-analysis was performed to search relevant studies using online databases EMBASE, PubMed and Web of Science published until February 1st, 2019. Ultimately, 65 studies met the inclusion criteria for this meta-analysis with 8.139 cases and 14.116 controls. The sensitivity, specificity, positive likelihood ratios (LR+), negative likelihood ratios (LR−), and other measures of PCA3 were pooled and determined to evaluate the diagnostic rate of PCa by the random-effect model.

**Results:**

With PCA3, the pooled overall diagnostic sensitivity, specificity, LR+, LR−, and 95% confidence intervals (CIs) for predicting significant PCa were 0.68 (0.64-0.72), 0.72 (0.68-0.75), 2.41 (2.16-2.69), 0.44 (0.40-0.49), respectively. Besides, the summary diagnostic odds ratio (DOR) and 95% CIs for PCA3 was 5.44 (4.53-6.53). In addition, the area under summary receiver operating characteristic (sROC) curves and 95% CIs was 0.76 (0.72-0.79). The major design deficiencies of included studies were differential verification bias, and a lack of clear inclusion and exclusion criteria.

**Conclusions:**

The results of this meta-analysis suggested that PCA3 was a non-invasive method with the acceptable sensitivity and specificity in the diagnosis of PCa, to distinguish between patients and healthy individuals. To validate the potential applicability of PCA3 in the diagnosis of PCa, more rigorous studies were needed to confirm these conclusions.

## INTRODUCTION

Prostate cancer (PCa) is a worldwide diagnosed malignant neoplasm, which has become the second mortality rate of tumors in elderly men (1-3). The clinic symptoms of PCa are mostly similar to benign prostatic hyperplasia (BPH), which makes a difficulty for clinician to accurately distinguish PCa from BPH (4). Due to lack of effective and timely diagnostic methods, the prognosis of PCa was generally poor (4). It is quiet important for clinicians to the detection of PCa at an early stage, in order to reduce the mortality of PCa, improve the survival rate and increase the opportunity of effective medical interventions (5-7).

Nowadays, serum prostate-specific antigen (PSA) is still widely used for PCa screening (5, 8). Serum PSA level has been widely used to detect PCa, which is an organ-specific antigen, but not a cancer-specific antigen (9). Several diseases, including BPH, prostatitis and PCa, might be associated with an elevated PSA level (5, 9). Though a high level of PSA is likely to be associated with PCa, the low specificity of PSA limits its use as a screening test and unnecessary biopsies (10). As a noninvasive diagnostic urine test, prostate cancer gene 3 (PCA3) is more accurate than PSA and can reduce the likelihood of false-positive results (11). Up to present, numerous individual studies have been performed to explore the diagnostic value of urine PCA3 in the management of PCa (12-18). However, these studies on the diagnostic performance of PCA3 have reported unclear or even conflicting results.

Based on a systematic review with meta—analysis, the objective of this study was to systematically collect the databases search results and perform an updated meta-analysis to assess the efficacy of diagnostic tests of PCA3 for the early detection of PCa.

## MATERIALS AND METHODS

### Literature search strategy

Studies were searched in the electronic databases EMBASE, PubMed and Web of Science up to February 1st, 2019. Available publications were identified using the following keywords or text words: ‘Differential Display clone 3’ or ‘DD3’ or ‘prostate cancer antigen 3’ or ‘PCA3’, ‘prostate cancer’ or ‘prostate neoplasms’ or ‘prostate carcinoma’ or ‘prostatic cancer’ or ‘prostatic neoplasm’ or ‘prostatic carcinoma’ or ‘cancer of prostate’ or ‘neoplasms of prostate’ or ‘carcinoma of prostate’, and ‘sensitivity’ or ‘specificity’ or ‘false negative’ or ‘false positive’ or ‘diagnosis’ or ‘detection’ or ‘accuracy’. For assessing all relevant studies, the most eligible literatures were retrieved. Moreover, relevant articles from reference lists of selected articles were searched to identify more relevant publications and avoid relevant information missing. No language restriction was applied.

There is no registered protocol for this systematic review. This systematic review and meta-analysis was conducted in accordance with the PRISMA guidelines, which compile guidelines for the reporting of meta-analysis of observational studies. The relevant studies included in this meta-analysis are previously published, and therefore, ethical approval and informed consent are not required.

### Criteria for inclusion and exclusion of published studies

The included studies must meet the inclusion criteria: (1) A case-control, nested case-control, or cohort randomized prospective or retrospective study, (2) Evaluate the diagnostic value of PCA3 in patients with PCa, (3) Available data for extraction to calculate sensitivity, specificity and other measures, (4) When duplications or the same patients used in several publications existed, the most recent or complete study was chosen in this meta-analysis. Additionally, the major exclusion criteria were as follows: (1) No available data; (2) Non-case-control studies, case reports, letters, reviewed editorial articles, (3) Duplicated publications with previous studies.

### Data extraction

The extracted appropriate information and data with a standard protocol were inspected by two researchers independently, to ensure the reliability and accuracy of the results. Moreover, the controversies were reviewed and settled through discussion by a third investigator, until all problems were finally resolved. The following information from each study were extracted: name of first author, publication date, country, ethnicity, mean age, PSA value (ng/mL), assay type, sample source, sample size, cut-off value, controls value (ng/ mL), PCa/non-PCa case, and raw data including true positive (TP), true negative (TN), false positive (FP), and false negative (FN) results.

In addition, the quality of each reference was also evaluated by two investigators independently, according to the revised QUADAS tools (19). Each domain contains seven questions, which can be answered by “yes”, “no” or “not clear” that assess the quality of included studies. An answer of “yes” means a low risk of bias, whereas “no” or “not clear” means a higher risk of bias in terms of the loss of some information from each literature.

### Statistical analysis

The statistical software STATA version 12.0 (StataCorp LP, College Station, TX) was performed to conduct all statistical data in this meta-analysis, and the Spearman test was used to analyze the threshold effect or the non-threshold effect. All of the statistical tests were two-sided, and P <0.05 was considered statistically significant. The pooled sensitivity, specificity, positive likelihood ratios (LR+), negative likelihood ratios (LR−), and the diagnostic odds ratio (DOR) as well as their corresponding 95% CIs were summarized to assess the diagnostic value of PCA3 in patients with PCa. Data were visualized as forest plots and receiver operating characteristic curves (ROC). The between-study heterogeneity was evaluated by Q test and I2 statistic, and P <0.05 was deemed statistically significant. As a quantitative measurement of inconsistency across different studies, I2-square value, ranged from 0 (no observed heterogeneity) to 100% (maximal heterogeneity), was also calculated. If the heterogeneity across studies was not identified, the fixed-effects model was used. Otherwise, the random-effects model was used in the meta-analysis. In addition, the summary receiver operating characteristic (sROC) curve was generated and the area under sROC curves (AUC) was calculated both overall and the subgroup analysis. Additionally, publication bias was investigated using Deek's funnel plot asymmetry test. When the P value of the Egger test was <0.05, the statistical significance was defined. Then, we replicated the funnel plot with its “missing” counterparts around the adjusted summary estimate.

## RESULTS

### Studies characteristics

**Figure 1:**
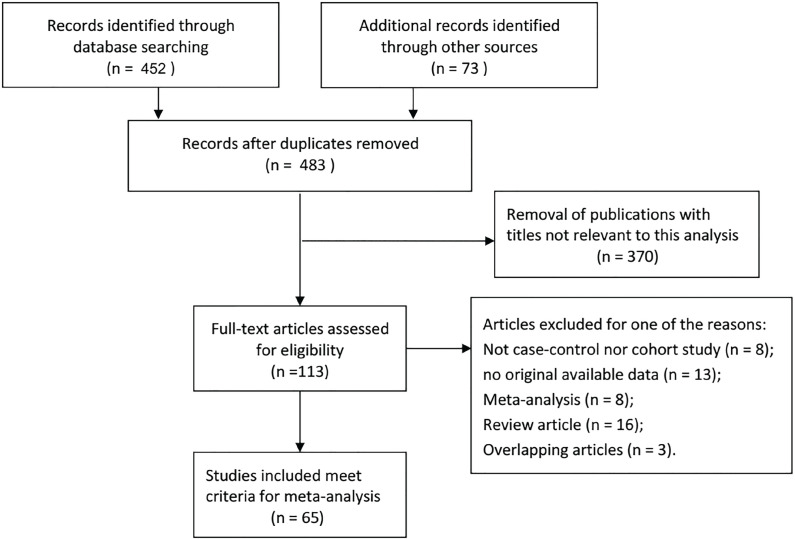
Flowchart of literature search and selection process.

As shown in Figure-1, 483 records were retrieved. After screening titles and abstracts of relevant articles, 418 articles were excluded because these were not related to the inclusion criteria. Finally, 65 case-control studies published between 2003 and 2018 were included in the meta-analysis (11-18, 20-76). All of these studies were retrospective in design.

The present meta-analysis included 8.139 cases and 14.116 controls from a total of 65 case-control studies about evaluating the diagnostic value of PCA3 in patients with PCa, and the detailed data of each study are listed in Table-1. Based on the studies described above, we retrieved data from 22.255 patients with PCA3 test and 5.065 patients with diagnosed PCa. All the studies presented the sensitivity, specificity, LR+, LR− and cut-off points. In these studies, these assay types, such as enzyme-linked immunosorbent assay (ELISA) and reverse transcription-polymerase chain reaction (RT-PCR), were applied to detect the expression level of PCA3. Besides, fifty studies were performed on Caucasian population, ten studies were conducted on Asian population, one study was carried out on African population, and the remaining studies involved more than one race.

**Table 1:** Characteristics and methodology assessment of individual studies included in the meta-analysis.

Year	First author	Country	Ethnicity	T Mean age (years)	T Mean PSA (ng/mL)	Assay type	Sample source	Cut-off value	Case	Control	TP	FP	FN	TN	QUADA
2018	Li	China/Asian	Asian	NR	NR	PCR	Urine	33.9	24	53	21	9	3	42	12
2017	Sanda MG	US	Caucasian/Asian/African	62 (33-85)	4.8 *(0.3-460.4)	PCR	Urine	20	264	262	104	18	160	244	10
2017	Zhou	China/Asian	Asian	65.3±7.8	7.1±1.77	PCR	Urine	23.5	33	89	27	48	6	41	11
2017	Rubio-Briones	Spain	Caucasian	61.7±6.12	4.49±1.99	PCR	Urine	35	161	396	115	186	46	210	11
2017	Bernardeau S	France	Caucasian	66.5	5.6	PCR	Urine	24	47	78	34	34	13	44	10
2017	Cao	US	Caucasian/African	63* (59–68)	NR	PCR	Urine	35	77	195	50	55	27	140	12
2017	Wang	China/Asian	Asian	45-92	NR	PCR	Urine	40.38	169	425	112	81	57	344	12
2016	Abdellaoui Maane I	Morocco	Caucasian	52-73	6.16-15.9	PCR	Tissue	cutoff 1.035	64	41	48	7	16	34	11
2016	Tan	China/Asian	Asian	71 (60-89)	32.4 (2.5-199.7)	LAMP	Serum	NR	89	101	76	8	13	93	10
2016	Nygård Y	Norway	Caucasian	64.0 (65.1*; 62.9-65.2^a^)	9.1 (7.2*;8.3-9.9^a^)	PCR	Urine	35	70	54	45	12	25	42	10
2015	Merola R	Italy	Caucasian	NR	NR	PCR	Urine	51	195	212	185	85	10	127	11
2015	Kaufmann	Germany	Caucasian	65 ± 5.6 (52-79)	10 ± 4.4 (4.0-25.0)	PCR	Urine	35	22	27	16	10	6	17	12
2015	Rubio-Briones	Spain	Caucasian	64(58-69)	5.2(4.3-7.2)	PCR	Urine	35	318	374	190	90	128	284	11
2015	Vlaeminck-Guillem V	France	Caucasian	64 ± 7(64*,59-69)	6.2 ± 4.3(6.6*,5-9.4)	PCR	Urine	35	480	535	326	155	154	380	10
2015	Coelho FF	Brasil	Caucasian	65.8±7.35	NR	PCR	Urine	cutoff 0.2219	22	37	14	9	8	28	12
2015	Huang	China/Asian	Asian	70*(51-88)	13.67(7.98–29.02)^b^	PCR	Urine	35	112	24	90	9	22	15	11
2014	Ruffion	France	Caucasian	63(58-67)^b^	5.9(4.7-7.9)^b^	PCR	Urine	35	274	321	173	90	101	231	11
2014	Nygård Y	Norway	Caucasian	54.0 ± 6.4; 65.1*	9.1 ± 4.7; 7.2*	PCR	Urine	35	59	65	42	18	17	47	10
2014	Wei	US	Caucasian/Asian/African	62±8	8±14	PCR	Urine	35	331	528	205	122	126	406	13
2014	Porpiglia	Italy	Caucasian	65 (60-70)^b^	6.9 (5.2-9.8)^b^	PCR	Urine	32.5	52	118	34	29	18	89	10
2014	Chevli	US	Caucasian	64.8± 9.2	6.4±23.3^c^	PCR	Urine	35	902	2171	478	543	424	1628	12
2013	Busetto	Italy/Rome	Caucasian	66.4 ± 5.3	6.8± 1.6	PCR	Urine	35	68	95	46	48	22	47	11
2013	Rubio-Briones	Spain	Caucasian	57.5±6.2 (57*, 40-74)	4.63±2.25 (4.04*, 0.37-19.5)	PCR	Urine	35	105	216	82	93	23	123	10
2013	Salagierski	Poland/Europe	Caucasian	66.2±6.8	7.5±1.9	PCR	Urine	35	24	56	18	24	6	32	11
2013	Ochiai	Japan	Asian	69*(42–89)	7.6 *(1.4–1908)	PCR	Urine	35	264	369	176	105	88	264	11
2013	Goode	US	Caucasian	66*(41–90)	4.8*(0.1–54.2)	PCR	Urine	35	95	361	48	116	47	245	11
2013	Stephan	Germany/Europe	Caucasian	65 *(41–81)	6.05 (0.50–19.77)	PCR	Urine	28	110	136	94	90	16	46	12
2012	Perdona	Italy/Europe	Caucasian	64.91±7.37	6.13 *(4.46–7.93)^b^	PCR	Urine	32.5	47	113	24	19	23	94	10
2012	Ng CF	China/Asian	Asian	71 (56-86)	20/10* (2-127)	PCR	Urine	35	17	24	12	2	5	22	12
2012	Crawford	US	Caucasian	64.4±8.6	8.0±20.0	PCR	Urine	35	802	1111	389	249	413	862	12
2012	Babera	Italy/Europe	Caucasian	64*	9.5*(3.7-28)	PCR	Urine	35	110	67	36	13	74	54	10
2012	Pepe	Italy/Europe	Caucasian	64*(48-74)	8.9*(4.5-10)	PCR	Urine	35	27	47	19	27	8	20	11
2012	Pepe	Italy/Europe	Caucasian	62.5*(48-72)	8.5 * (3.7-24)	PCR	Urine	35	32	86	23	50	9	36	11
2012	Sciarra	Italy/Europe	Caucasian	63.7±7.24	6.98±2.86	PCR	Urine	35	55	113	41	30	14	83	10
2012	Wu	US	Caucasian	63.5±7.4	11.0±8.5	PCR	Urine	35	46	57	18	13	28	44	11
2011	Vlaeminck-Guillem V	France	Caucasian	63 ± 7	6.2 ± 4.3	PCR	Urine	35	126	114	76	37	50	77	11
2011	Ochiai	Japan	Asian	66*(44-87)	7.2*(3.3-720.6)	PCR	Urine	35	35	67	26	17	9	50	11
2011	De La Taille A	France/Germany/Europe	Caucasian	63.0± 7.6	5.9 ± 2.1	PCR	Urine	35	207	309	133	74	74	235	12
2011	Adam	South Africa	African	67(35–89)	NR	PCR	Urine	35	44	61	34	30	10	31	11
2010	Cao	China/Asian	Asian	NR	NR	PCR	Urine	AUC:0.73	86	45	82	24	4	21	10
2010	Roobol	Netherlands/Europe	Caucasian	70.07(63.7–74.0)	2.74 (0.2–23.0)	PCR	Urine	35	122	599	83	265	39	334	11
2010	Rigau	Spain/Europe	Caucasian	65.7 (44–85)	11.86 (1.5–189)	PCR	Urine	35	73	142	50	58	23	84	12
2010	Auprich	France/Germany/Europe	Caucasian	63(35–90)	7.3(1–82.7)	PCR	Urine	35	255	366	164	110	91	256	12
2010	Ouyang	US	Caucasian	NR	NR	PCR	Urine	19	43	49	31	20	12	29	10
2010	Henderson	England/The Netherlands	Caucasian	69.9	10.1(3.03-44.2)	PCR	Urine	35	6	44	5	18	1	26	11
2010	Aubin	US	Caucasian	NR	(0.30-33.9)	PCR	Urine	35	190	882	92	189	98	693	12
2010	Morote	Spain	Caucasian	64* (39–85)	6.4*(1.5–189)	PCR	Urine	NR	83	161	75	34	8	127	11
2010	Nyberg	Sweden/Europe	Caucasian	63 *(57–70)^b^	7.9 *(5.1–12.8)^b^	PCR	Urine	35	18	44	12	24	6	20	10
2010	Shen	China/Asian	Asian	70.3(51–86)	NR	PCR	Urine	cutoff 0.107	35	64	22	6	13	58	10
2010	Schilling	Germany/Europe	Caucasian	NR	7.7*(2.0–46.9)	ELISA	Urine	35	18	14	17	9	1	5	10
2009	Shappell	US	Caucasian	NR	NR	PCR	Urine	35	11	19	8	3	3	16	11
2009	Wang	US	Caucasian	62 ±8.3(44-86)	8.7±12.4	PCR	Urine	35	87	100	46	20	41	80	10
2009	Mearini	Italy/Europe	Caucasian	69.1(53–83)	1.08-172.0	PCR	Urine	AUC: 0.814	70	26	42	0	28	26	10
2008	Haese	Europe	Caucasian	64.4±6.6	8.9 ± 7.6	PCR	Urine	35	128	335	60	94	68	241	11
2008	Deras	US/Canada	Caucasian	64 (32–89)	7.8 (0.3–484)	PCR	Urine	35	206	357	111	93	95	264	12
2008	Nakanishi	US	Caucasian/African	60 (45–70)	5.7 (1.0–27.0)	PCR	Urine	25	40	102	25	19	15	83	12
2008	Laxman	US	Caucasian	NR	NR	PCR	Urine	AUC:0.66	138	96	91	23	47	73	11
2007	Marks	US/Canada	Caucasian	64 ± 7(64*45-83)	7.4 ± 4.3(6.1*2.5-31.1)	PCR	Urine	35	60	166	35	46	25	120	11
2007	Van Gils MPMQ	Netherlands/Europe	Caucasian	64.3±7.2	7.49 ± 2.93	PCR	Urine	58	174	360	113	122	61	238	12
2007	Van Gils MPMQ	Netherlands/Europe	Caucasian	64±7.2	8.73± 6.61	PCR	Urine	43	23	44	14	9	9	35	10
2007	Van Gils MPMQ	Netherlands/Europe	Caucasian	NR	NR	PCR	Urine	66	23	44	15	8	8	36	10
2006	Groskopf	US	Caucasian	67±11 (45-93)	7.7±14.1(0.4-101.7)	PCR	Urine	cutoff 0.05	16	52	11	11	5	41	12
2004	Tinzl	Austria/Europe	Caucasian	64.7 (41-89)	0.59 -1486	PCR	Urine	cutoff 0.5	79	122	65	29	14	93	13
2004	Fradet	Canada	Caucasian	64* (40-87)	0.1-144	PCR	Urine	cutoff 0.5	152	291	100	32	52	259	12
2003	Hessels	Netherlands/Europe	Caucasian	NR	NR	PCR	Urine	cutoff 0.2	24	84	16	14	8	70	11

* median;

a 95%CI;

b IQR (interquartile range);

c missing;

d SEM;

AUC area under curve;

PCA3/PSA

NA data are not available; mean median (ranges); cutoff values were not provided because these studies found serum PCA3 has no correlation with PCa.

### Quantitative synthesis results

**Figure 2:**
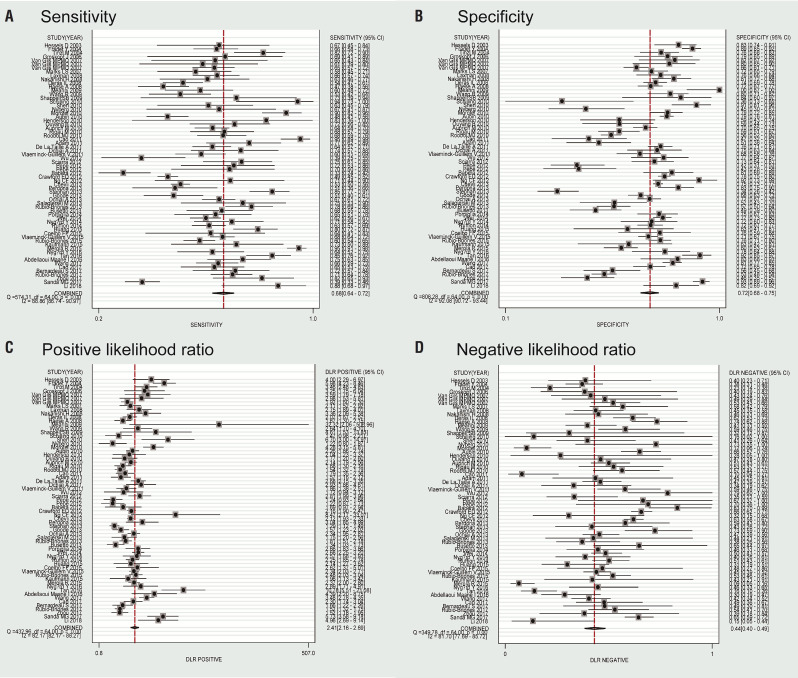
Flowchart of literature search and selection process.

**Figure 3:**
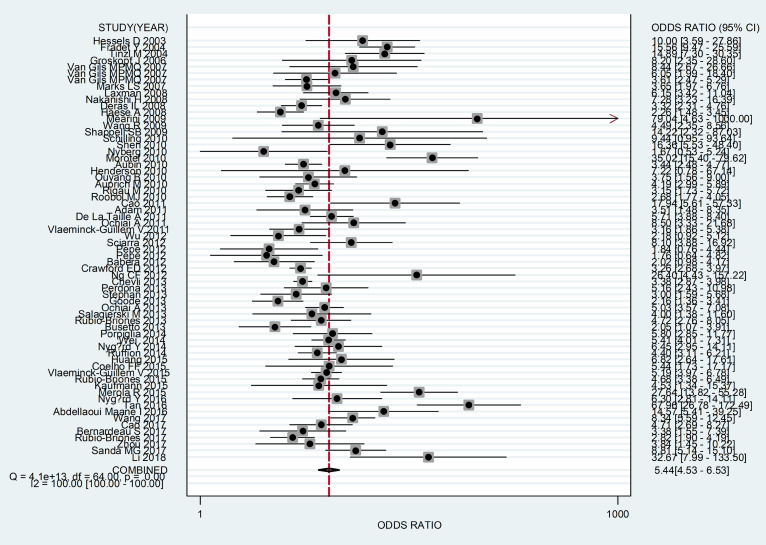
Forest plots of summary diagnostic odds ratio of by PCA3 as a diagnostic marker for PCa in this meta-analysis. Each solid circle represents an eligible study. The size of solid circle reflects the sample size of each eligible study. Error bars represent 95% CIs.

**Figure 4:**
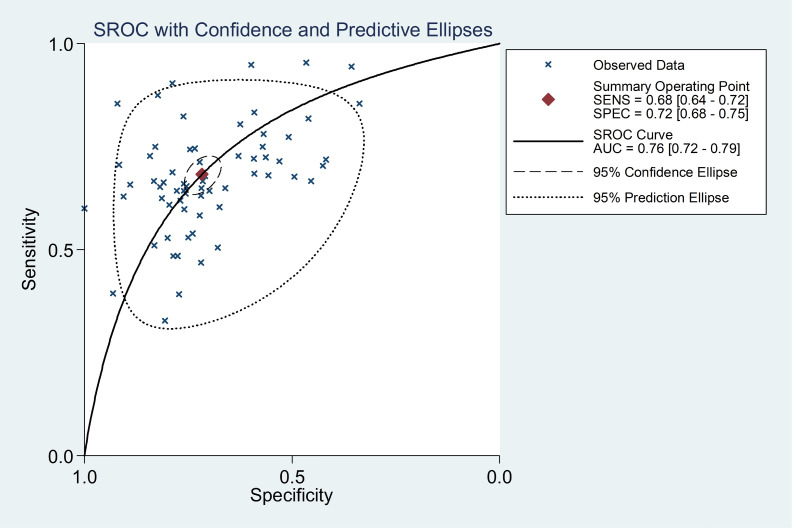
Summary receiver operating characteristic curves of PCA3 for the diagnosis of PCa. Each solid circle represents an eligible study. The size of solid circle represents the sample size of each eligible study. The overall diagnostic efficiency is summarized by the regression curve.

In this meta-analysis, the random-effects model was selected to calculate the sensitivity, specificity, LR+, and LR− with corresponding 95% CIs, because of the obvious between-study heterogeneity among those studies (P <0.05). The meta—analytic results showed that the pooled overall diagnostic sensitivity, specificity, LR+, LR− and 95% CIs about PCA3 for predicting significant PCa were 0.68 (0.64-0.72), 0.72 (0.68-0.75), 2.41 (2.16-2.69), 0.44 (0.40-0.49), respectively (Figure-2). Moreover, the summary diagnostic odds ratio (DOR) and 95% CIs for the diagnostic value of PCA3 in PCa patients was 5.44 (4.53-6.53) (Figure-3). In addition, AUC and 95% CI was 0.76 (0.72-0.79) (Figure-4).

### Test of heterogeneity

The I2-square of sensitivity, specificity, LR+, LR− and DOR in this meta-analysis were as follows: 88.86%, 92.08%, 82.17%, 81.70% and 100%, which proved that the heterogeneity between eligible studies was significant. As a result, the random effects model was chosen to synthesize the relevant data mentioned above.

### Publication bias

**Figure 5:**
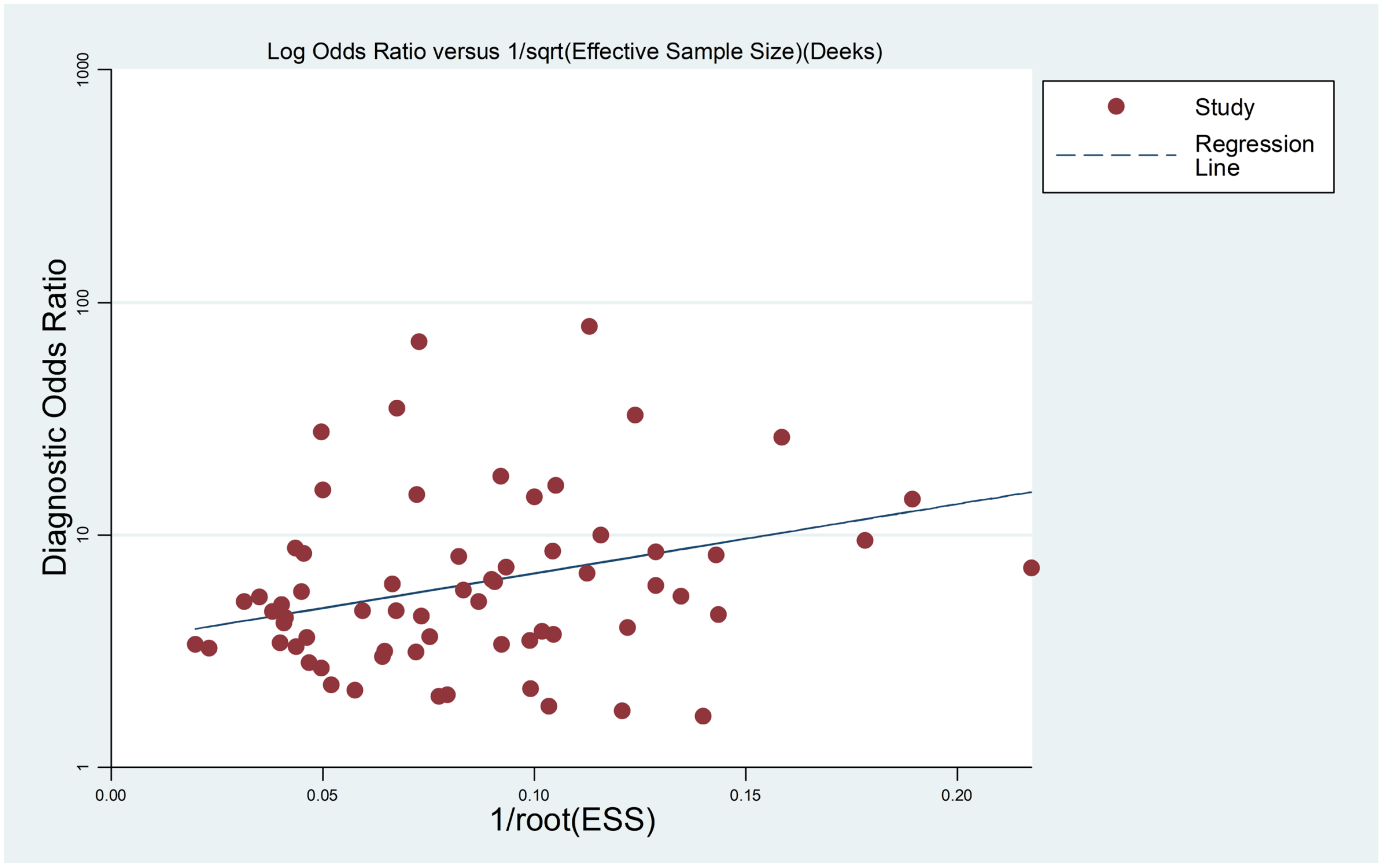
Linear regression test of funnel plot asymmetry. The statistically non-significant P value of the slop coefficient indicates symmetry of the data and a low likelihood of publication bias.

The potential publication bias of the included studies was evaluated through the Deek's funnel plot asymmetry test. The data of the slope coefficient of the regression line were symmetric, which suggested that the meta-analysis did not have a likelihood of publication bias (Figure-5).

## DISCUSSION

Though PCa presents a slow progress, it has become a big threat to the health of men (4). Thus, the intervention at the early staging of PCa improves clinical prognosis. Serum PSA, DRE and transrectal ultrasound are still served as the screening of PCa in many countries and areas, which provides clinicians a low positive rate in the diagnosis of PCa (8). Among them, PSA is a serum marker widely used for screening of PCa in past years (4, 7). However, the proportion of positive biopsy is less than 50% in men with elevated serum PSA (10, 77). Therefore, the false-positive of PSA results may lead to unnecessary prostate biopsies and cause the complications of prostate biopsy (78). For these reasons, the searching for novel specific biomarkers of PCa has been attempted all the time.

In recent years, several serologic and pathologic biomarkers, with higher specificity than serum PSA, have been found to reduce unnecessary biopsy and inform the treatment (79, 80). Among them, PCA3 is one of the most valuable biomarkers in the detection of PCa (80). There are different expression of PCA3 gene in PCa tissue and other noncancerous tissue, which provides a great help for clinician to distinguish PCa from other prostatic diseases (80, 81). PCA3 gene is located on the long arm of chromosome 9 with 23kb long of nucleic acid and four exons and it cannot be translated into protein in normal cells (11, 82). In addition, it is a specific biomarker, over-expressed in more than 95% of PCa cells, so it can help to distinguish benign from cancerous prostate cells with an accuracy approaching 100% (83). Besides, PCA3 is also not affected by age, prostate volume or other prostatic diseases (81). In clinic, it is normally extracted in urine samples collected after DRE (11). And PROGENSA PCA3 assay has been already widely used to measure the level of urinary PCA3, and it can also been measured in serum and tissue samples (20, 23, 84).

Over the past years, many studies have increased to evaluate the value of PCA3 in the detection of PCa. In order to elucidate the expression differences of PCA3, meta-analysis has been updated to comprehensively and systematically investigate the diagnosis accuracy of PCA3 level in PCa patients. However, the outcomes of these studies remained inconsistent and controversial. There were several variables in these studies, such as the different ethnicities, the small sample size of individual study, the possible limited effect of individual patient data, among other factors, which could have caused the limited statistical power in the published studies. Compared with previous review and meta—analysis (85-87), this meta-analysis contains more studies for the sake of the sufficient evidence of our results. Furthermore, the publication of the previous meta-analysis might generate great influence on the results. All these factors made contributions to the development of the current meta-analysis.

Compared to a single study, meta-analysis would provide more sufficient results. Thus, we suggested that there existed stronger advantages to prove the relevance between the level of PCA3 and the diagnosis of PCa. Though it was deemed that PCA3 might be a valuable diagnostic biomarker of PCa in the previous studies, correlation between PCA3 level and the diagnosis of PCa remains unclear. Therefore, we need a better method for further analysis and elaboration about the diagnostic value of PCA3 for PCa. In the present meta-analysis, the summary DOR and 95% CIs for PCA3 was 5.44 (4.53-6.53), and AUC and 95% CIs was 0.76 (0.72-0.79). Thus, the above results revealed that PCA3 could be acceptable as a valuable biomarker to distinguish PCa patients from healthy individuals.

Overall, the sufficient statistical evidences including the large sample size were used to estimate the diagnostic value of PCA3 in the detection of PCa. However, several limitations were involved in this meta-analysis. First of all, the ethnicities involved in these studies were mainly Caucasians, However, Asian and African populations were included in relatively few studies. Thus, more attention should be paid to the influence of ethnicity. Secondly, there was a threshold effect and obvious heterogeneity in this meta-analysis, probably due to the large difference in reagent resource, patient characteristics, the assay type and the cut-off value. Moreover, the lack of sufficient data, the internal references and cut-off values were not considered in meta-regression analysis. Hence, it might reduce the reliability of our meta-analysis. In addition, more attention should be paid in further researches to the comparison of PCA3, PSA, and other biomarkers in the diagnosis of PCa. To improve reliability of the meta-analysis, well-designed studies with large sample size should be continued to evaluate the effectiveness of PCA3 in the detection of PCa in the subsequent years.

## CONCLUSIONS

This meta-analysis suggested that PCA3 is acceptable as a valuable diagnostic biomarker in the management of PCa, which is a non-invasive method with the acceptable sensitivity and specificity in the diagnosis of PCa to distinguish patients from healthy individuals. To further evaluate the diagnostic value of PCA3 in patients with PCa, more well-designed studies with large sample sizes are needed to validate the effectiveness of PCA3 to differentially diagnose PCa.

## Abbreviations

PCA3: prostate cancer antigen 3;
PCa: prostate cancer;
LR+: positive likelihood ratios;
LR−: negative likelihood ratios;
CIs: confidence intervals;
sROCs: ummary receiver operating characteristic;
AUC: area under sROC curves;
BPH: benign prostatic hyperplasia;
PSA: prostate-specific antigen;
TP: true positive;
TN: true negative;
FP: false positive;
FN: false negative;
DOR: diagnostic odds ratio;
ELISA: enzyme-linked immunosorbent assay;
RT-PCR: reverse transcription-polymerase chain reaction.
